# The origin and diversification of pteropods precede past perturbations in the Earth’s carbon cycle

**DOI:** 10.1073/pnas.1920918117

**Published:** 2020-09-24

**Authors:** Katja T. C. A. Peijnenburg, Arie W. Janssen, Deborah Wall-Palmer, Erica Goetze, Amy E. Maas, Jonathan A. Todd, Ferdinand Marlétaz

**Affiliations:** ^a^Plankton Diversity and Evolution, Naturalis Biodiversity Center, 2300 RA Leiden, The Netherlands;; ^b^Department Freshwater and Marine Ecology, Institute for Biodiversity and Ecosystem Dynamics, University of Amsterdam, 1090 GE Amsterdam, The Netherlands;; ^c^Department of Oceanography, University of Hawai’i at Mānoa, Honolulu, HI 96822;; ^d^Bermuda Institute of Ocean Sciences, St. Georges GE01, Bermuda;; ^e^Department of Earth Sciences, Natural History Museum, London SW7 5BD, United Kingdom;; ^f^Centre for Life’s Origins and Evolution, Department of Genetics, Evolution and Environment, University College London, London WC1E 6BT, United Kingdom;; ^g^Molecular Genetics Unit, Okinawa Institute of Science and Technology, Onna-son 904-0495, Japan

**Keywords:** plankton, ocean acidification, phylogenomics, fossil record, calcification

## Abstract

Pteropods are a group of planktonic gastropods that are widely regarded as biological indicators for assessing the impacts of ocean acidification. Their aragonitic shells are highly sensitive to acute changes in ocean chemistry. However, to gain insight into their potential to adapt to current climate change, we need to accurately reconstruct their evolutionary history and assess their responses to past changes in the Earth’s carbon cycle. Here, we resolve the phylogeny and timing of pteropod evolution with a phylogenomic dataset (2,654 genes) incorporating new data for 21 pteropod species and revised fossil evidence. In agreement with traditional taxonomy, we recovered molecular support for a division between “sea butterflies” (Thecosomata; mucus-web feeders) and “sea angels” (Gymnosomata; active predators). Molecular dating demonstrated that these two lineages diverged in the early Cretaceous, and that all main pteropod clades, including shelled, partially-shelled, and unshelled groups, diverged in the mid- to late Cretaceous. Hence, these clades originated prior to and subsequently survived major global change events, including the Paleocene–Eocene Thermal Maximum (PETM), the closest analog to modern-day ocean acidification and warming. Our findings indicate that planktonic aragonitic calcifiers have shown resilience to perturbations in the Earth’s carbon cycle over evolutionary timescales.

## Significance

Pteropods are abundant aragonitic calcifiers, contributing up to 89% of total pelagic calcification. Because of their delicate shells, they are considered “canaries in the coalmine” to indicate impacts of ocean acidification. Their sensitivity to high CO_2_ levels and limited fossil record has led to the widely held view that pteropods only became abundant after the PETM. Based on phylogenomic analyses, we show that all major pteropod groups have Cretaceous origins and, hence, they must have survived past perturbations in the Earth’s carbon cycle. Although this suggests that pelagic aragonitic calcifiers have been more resilient to past ocean acidification than currently thought, it is unlikely that pteropods have experienced global change of the current magnitude and speed during their evolutionary history.

**Fig. 1.**
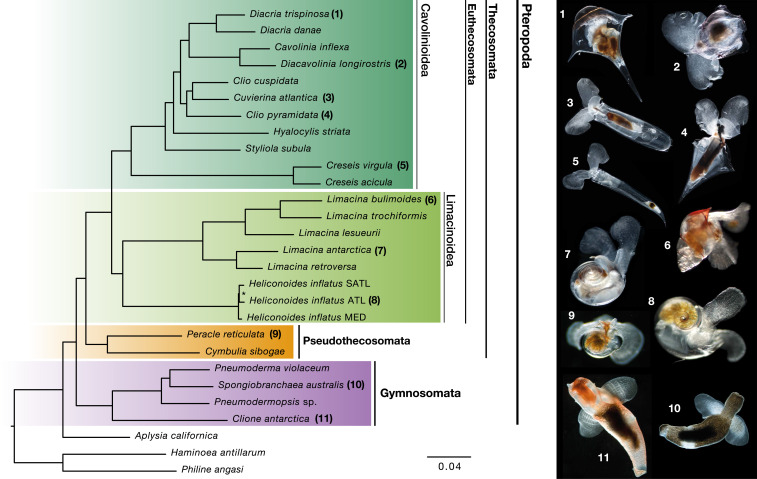
Phylogenomics resolves evolutionary relationships of pteropods. Euthecosomes (fully shelled species) and pseudothecosomes (ranging from fully shelled to unshelled species) are now recovered as sister clades in our molecular analysis restoring the Thecosomata as a natural group. Thecosomata (sea butterflies) and Gymnosomata (sea angels) are sister clades congruent with traditional morphology-based views. The superfamilies Cavolinioidea with uncoiled shells and Limacinoidea with coiled shells are also recovered as monophyletic sister clades. Maximum likelihood phylogeny of 25 pteropod taxa, plus 3 outgroups assuming an LG+Γ model. The dataset comprises 2,654 genes, concatenated as 834,394 amino acid positions with 35.8% missing data. A nearly identical topology is obtained modeled under CAT-GTR+Γ with a reduced dataset of 200 genes (*SI Appendix*, Fig. S1). Higher taxonomic divisions are indicated. All nodes receive maximal bootstrap support except the node with an asterisk (bootstrap 95%). (*Right*) Images of living pteropod species (numbers correspond with taxon labels; not to scale) collected and photographed by the authors. Scale bar indicates substitutions per site.

Pteropods are marine gastropods that spend their entire life in the open water column. A remarkable example of adaptation to pelagic life, these mesmerizing animals have thin shells and a snail foot transformed into two wing-like structures that enable them to “fly” through the water column (Fig. 1). Pteropods are a common component of marine zooplankton assemblages worldwide, where they serve important trophic roles in pelagic food webs and are major contributors to carbon and carbonate fluxes in the open ocean (1–5).

Shelled pteropods have been a focus for global change research because they make their shells of aragonite, a metastable form of calcium carbonate that is 50% more soluble than calcite (6, 7). As their shells are susceptible to dissolution, pteropods have been called “canaries in the coal mine,” or sentinel species that signal the impacts of ocean acidification on marine calcifiers (e.g., refs. 8 and 9). Although shelled pteropods are already negatively affected in several regions of the global ocean (e.g., refs. 10–12), and will likely be seriously threatened if CO_2_ levels continue to rise (e.g., refs. 13–15), little is known about the evolutionary history of the group.

Improving the phylogenetic framework for pteropods and estimating the timing of divergence for major lineages will help determine the effects of past periods of high atmospheric CO_2_, such as the Paleocene–Eocene Thermal Maximum (PETM; 56 million years ago [Ma]), on pteropod diversification and survivorship. The PETM is widely regarded as the closest geological analog to the modern rise in ocean-atmosphere CO_2_ levels, global warming, and ocean acidification (16–18). Knowing whether major pteropod lineages have been exposed during their evolutionary history to periods of high CO_2_ is important to extrapolate from current experimental and observational studies to predictions of species-level responses to global change over longer timescales.

Pteropods are uniquely suited to shed light on long-term marine evolutionary dynamics because they are the only living metazoan plankton with a good fossil record (19). The only other pelagic groups with abundant fossil records are protists, including foraminifers, radiolarians, coccolithophores, and extinct animal lineages, such as ammonites. Although the earliest pteropod, a single internal mold of *Heliconoides* sp., is described from the Campanian (∼72 Ma; ref. 20), pteropods are generally considered to have Paleogene origins (21–23). This inference is because of the long temporal gap until abundant pteropod fossils are found, which is from the Eocene onward (∼56 Ma; reviewed in ref. 24). The fossil record of pteropods is also limited because their shells are very thin and are only preserved in waters above the aragonite saturation depth, which is shallower than the saturation depth of calcite (25). In addition, several groups of pteropods have only partial shells or are shell-less as adults and thus are rarely preserved in marine sediments. Hence, resolving the evolutionary history of pteropods requires a combination of molecular and fossil-based approaches to assess past diversification and timing.

While most researchers recognize pteropods as composed of two orders, Thecosomata (“sea butterflies”) and Gymnosomata (“sea angels”), a recent classification (26) identifies three suborders: (*i*) Euthecosomata, fully-shelled, omnivorous mucus-web feeders; (*ii*) Pseudothecosomata, a poorly known group with shelled, partially-shelled, and unshelled species that also use mucus webs for feeding; and (*iii*) Gymnosomata, with shell-less adults that are specialized predators, primarily on euthecosomes. Progressive evolution toward loss of shells as an adaptation to planktonic life has been proposed for the group (21, 27), but never fully tested.

Previous attempts to resolve the molecular phylogeny of pteropods have relied on small subsets of genes, and resolution has been limited, especially at deeper nodes, due to large rate heterogeneity and insufficient taxonomic signal (28–31). Here, we used a phylogenomic approach based on transcriptome sequencing to fully resolve the phylogeny of pteropods. Using the pteropod fossil record to calibrate the timing of divergence, we estimate that two major groups of pteropods, sea butterflies and sea angels, diverged in the early Cretaceous, and thus both groups must have survived previous global perturbations to the ocean’s carbonate system.

## Results and Discussion

### Robust Phylogenomic Resolution.

We generated transcriptome data for 21 pteropod species collected along two basin-scale transects in the Atlantic Ocean. The number of individuals used for RNA extraction varied from 1, in most cases, to 10 (*SI Appendix*, Tables S1 and S2). We also incorporated available data for three additional pteropod species (13, 15, 32) and three outgroup species: the sea hare *Aplysia californica*, within the proposed sister group of pteropods (Aplysiida), and two members of Cephalaspidea, *Haminoea antillarum* and *Philine angasi*, from ref. 32. Our taxonomic sampling included representatives of all extant families of Euthecosomata, two of three families of Pseudothecosomata, and two of six families of Gymnosomata. All superfamilies of Pteropoda were sampled except Hydromyloidea (Gymnosomata).

We inferred a set of single-copy orthologs using gene family reconstruction from assembled and translated transcripts and selected 2,654 single-copy nuclear genes for phylogenetic inference based on their taxonomic representation. These selected genes are well represented in our transcriptomes, with a median of 1,815 genes per species and the least being 682 genes (77.4% missing data) for *Diacria trispinosa* (*SI Appendix*, Table S2). We combined these single-copy orthologs in a large data matrix of 834,394 amino acids with 35.75% missing data.

Using the large data matrix (2,654 genes) and a site-homogeneous model of evolution (LG+Γ_4_), we recovered a fully resolved phylogenetic tree with maximal support values at all interspecific nodes (Fig. 1). To account for the putative limitations of site-homogeneous models that could lead to systematic error, we also applied a site-heterogeneous model (CAT + GTR+Γ) using Bayesian inference. For this analysis, we used a reduced data matrix comprised of the 200 most informative genes (108,008 amino acids; *Materials and Methods*), and we recovered an identical topology except for a single terminal node (*SI Appendix*, Fig. S1).

### Pteropod Systematics Reappraised.

Our trees confirm that pteropods are a monophyletic group with sea hares (*Aplysia*) as their closest sister group (28, 30). Pteropods are split into two sister clades: Thecosomata and Gymnosomata, which is congruent with traditional classification but strongly supported by molecular evidence in the present study. An obvious shared character for pteropods are the wing-like structures or “parapodia” used for swimming. Histological and ultrastructural studies showed that the muscle arrangements in parapodia are very complex and look similar in Thecosomata and Gymnosomata, supporting a homologous origin (28). Thecosomata comprise the Euthecosomata and Pseudothecosomata clades, whose member species are all omnivorous mucus-web feeders (33). Their common feeding mechanism is reflected in the well-developed mucus-secreting pallial gland that is shared among all thecosomes, as well as a muscular gizzard with which they can crush the hard exoskeletons of their prey (21). Gymnosomata are shell-less at the adult stage and are specialized carnivorous hunters. They have several morphological characters that set them apart from Thecosomata, including tentacle-like structures called “buccal cones” and hook sacs to grab and manipulate shelled pteropod prey (28, 34). Hence, the recent revision by Bouchet et al. (26) with three separate suborders (Euthecosomata, Pseudothecosomata, Gymnosomata) should revert back to the original classification of the order Pteropoda with two main clades: suborder Thecosomata, comprising the Euthecosomata and Pseudothecosomata; and suborder Gymnosomata, as shown in Fig. 1. Within the fully-shelled Euthecosomata, we obtained maximal support for the coiled Limacinoidea and uncoiled Cavolinioidea superfamilies as sister clades. These results finally stabilize the higher-level taxonomy of the group, which has been debated ever since Cuvier (1804; ref. 35) established the Pteropoda as a separate order of molluscs.

In agreement with previous molecular phylogenetic analyses (30, 31), we find good support for lower-level groupings (e.g., genera *Diacria* and *Limacina*) and recover *Creseis* as the earliest diverging lineage within the uncoiled shelled pteropods. The genus *Clio*, however, is paraphyletic in our analyses, with *Clio cuspidata* and *Cuvierina atlantica* grouping together and *Clio pyramidata* either as a sister taxon to this group (maximum-likelihood tree; Fig. 1) or to the clade *Cavolinia* + *Diacavolinia* + *Diacria* (Bayesian tree; *SI Appendix*, Fig. S1). These results are congruent with the branching obtained using broader taxon sampling but only three genes (31). Thus, it seems plausible that the genus *Clio* consists of two distinct groups, which could be characterized by distinct larval shell shapes, including *C. cuspidata* and *Clio recurva* in one clade and *C. pyramidata* and *Clio convexa* in the other. Sampling of additional species for transcriptome sequencing and more detailed morphological analysis are necessary to definitively revise the taxonomy of this genus.

### Divergence Times of Major Pteropod Lineages.

**Fig. 2.**
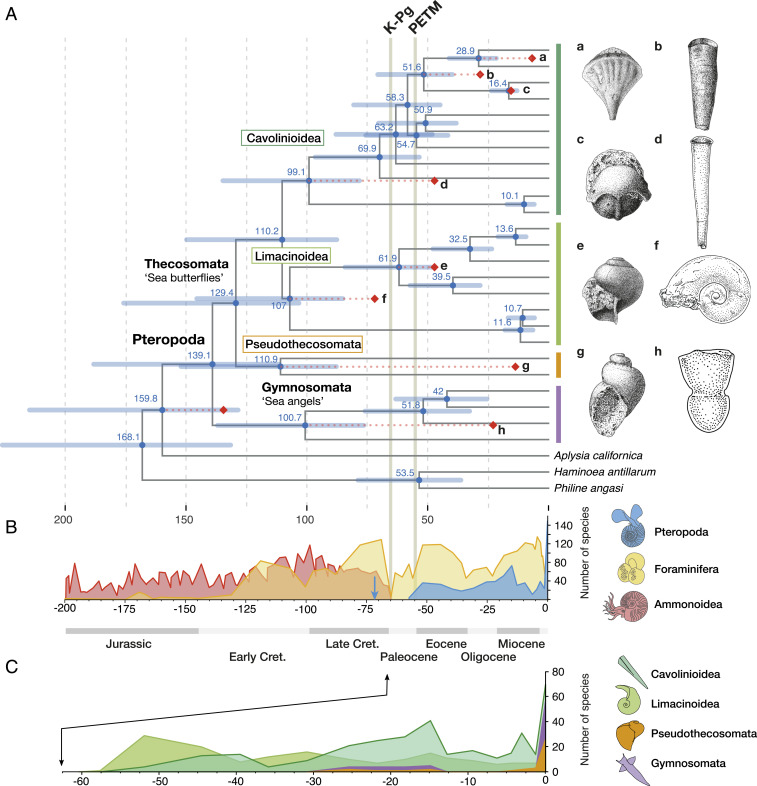
All major lineages of pteropods diverged in the Cretaceous and thus must have survived previous major global change events. (*A*) Chronogram of pteropods (25 taxa and 3 outgroups) based on 200 genes, concatenated as 108,008 amino acid positions, analyzed under a CAT-GTR+Γ evolutionary model and CIR relaxed clock model with a birth–death prior on divergence times and a gamma prior on the age of the root (150 ± 70 Ma). Red diamonds indicate fossil-calibrated nodes, with red dotted lines to show divergence from minimum ages of the fossils. (*Right*) Drawings of fossil species used for the calibrations of nodes: a, *Diacria sangiorgii*; b, *Vaginella gaasensis*; c, *Cavolinia microbesitas*; d, *Euchilotheca ganensis*; e, *Limacina gormani*; f, *Heliconoides* sp.; g, *P*. *amberae*; and h, *Clione ? imdinaensis* (Table 1). The outgroup calibration is *A*. *neocomiensis* (no drawing available). Two major global change events are indicated as K–Pg (Cretaceous–Paleogene asteroid impact; 66 Ma) and PETM (Paleocene–Eocene Thermal Maximum; ∼56 Ma). (*B*) Curves for ammonite (red), planktonic foraminifer (yellow), and pteropod (blue) observed species diversity through time are based on sediment records from refs. 44 and 46 and our own database (Dataset S1), respectively. The blue arrow indicates the oldest known pteropod fossil. (*C*) More detailed records of observed species diversity through time for different pteropod groups: Cavolinioidea and Limacinoidea (green), Pseudothecosomata (orange), and Gymnosomata (purple).

Estimating divergence times based on genome-scale datasets has been shown to be accurate and powerful; however, this depends on the use of realistic evolutionary and clock models as well as reliable fossil calibration schemes (36–38). We inferred divergence times of the main pteropod lineages based on a thorough revision of their fossil record (ref. 24 and Dataset S1). This revision provided eight meaningful calibrations, which were applied as minimum ages based on the oldest representatives for different clades of pteropods (Table 1 and Fig. 2*A*) (39–44). Within the sister clade of Pteropoda (Aplysiida), the shelled genus *Akera* has by far the best and oldest known fossil record (42, 45). We reassessed the fossil record of *Akera* and chose the oldest confidently identified species (*Akera neocomiensis*) to provide the calibration for the Aplysiida + Pteropoda clade (Table 1). However, as there is some uncertainty concerning the earliest occurrence of *Akera* and the phylogenetic placement of the *Heliconoides* sp. calibration, we tested five additional calibration schemes to assess the robustness of our analyses (*Materials and Methods* and *SI Appendix*). To ensure accurate reconstructions, we chose a realistic model of sequence evolution (CAT-GTR) using the reduced data matrix of 200 genes, with a birth–death prior on divergence times and a gamma prior on the age of the root. We performed cross-validation to select the best clock model (CIR) and prior parameters (birth–death; Fig. 2*A*), and also verified that the divergence times reported under alternative choices were not markedly different for the major clades (Table 2 and *SI Appendix*, Figs. S2–S4).

**Table 1.** Nine fossil calibrations used in the molecular dating analyses as minimum ages with soft bounds

Calibrated node	Fossil species	Epoch: stage	Minimum age, Ma	Ref.
a: *Diacria*	*Diacria sangiorgii*	Miocene: Tortonian	7.2	39
b: *Cavolinia*+*Diacavolinia*+*Diacria*	*Vaginella gaasensis*	Oligocene: Rupelian	28.1	40
c: *Cavolinia*+*Diacavolinia*	*Cavolinia microbesitas*	Miocene: Late Burdigalian	16	41
d: Cavolinioidea	*Euchilotheca ganensis*	Eocene: Ypresian	47.8	43
e: *Limacina*	*Limacina gormani*	Eocene: Ypresian	47.8	40
f: Limacinoidea	*Heliconoides* sp.	Cretaceous: Campanian	72.1	20
g: Pseudothecosomata	*Peracle amberae*	Oligocene: Chattian–Burdigalian	16	41
h: Gymnosomata	*Clione ? imdinaensis*	Oligocene: Chattian	23	41
i: Pteropoda+*Aplysia*	*Akera neocomiensis*	Cretaceous: Early Hauterivian	133	42

Letters refer to nodes labeled a to i in the chronogram of Fig. 2*A*. Ages are given in million years ago (Ma).

**Table 2.** Inferred divergence times of key pteropod clades under six calibration schemes, the CIR relaxed clock model, and birth–death priors based on 200 genes

Calibration scheme	Pteropoda	Thecosomata	Euthecosomata	Pseudothecosomata	Gymnosomata
s1: *Akera* 163.1 Ma/Euthecosomata	147.5 (121–195)	133.7 (109–177)	112.1 (92–148)	108.0 (80–144)	91.8 (64–132)
s2: *Akera* 133 Ma/Euthecosomata	142.5 (112–190)	129.6 (101–174)	108.2 (85–146)	104.6 (75–145)	91.4 (63–133)
s3: *Akera* 163.1 Ma/Limacinoidea	149.4 (119–200)	134.6 (107–181)	112.5 (89–153)	111.6 (82–151)	87.3 (62–123)
s4: *Akera* 133 Ma/Limacinoidea	139.1 (111–188)	129.4 (103–176)	110.2 (88–150)	110.9 (88–152)	100.7 (76–137)
s5: no *Akera*/Euthecosomata	135.4 (100–193)	125.4 (92–180)	107.2 (79–154)	105.5 (75–152)	90.1 (62–140)
s6: no *Akera*/Limacinoidea	141.9 (106–193)	131.5 (98–180)	111.3 (84–155)	110.3 (79–154)	99.9 (65–143)

Calibration schemes 1 to 6 (s1 to s6) differ in terms of the *Akera* calibration and the position of *Heliconoides* sp. as either the oldest Euthecosomata or Limacinoidea (*Materials and Methods*). Ages are given in million years ago (Ma), with 95% credibility intervals in brackets (see also Fig. 2*A*).

Our phylogenomic time-tree dates the origin of pteropods to the early Cretaceous (139.1 Ma with 95% credibility interval [CrI] 188.2 to 111.4; Fig. 2*A*), which is substantially older than the estimates based on previous molecular phylogenies (Paleocene and late Cretaceous as estimated by refs. 30 and 31, respectively). Choosing alternative calibration schemes, priors, or clock models increases the variance around this estimate, but results still place the origin of pteropods firmly in the early Cretaceous (Table 2 and *SI Appendix*, Figs. S2–S4). Previous phylogenetic studies (30, 31) employed only two and three genetic markers, respectively, and failed to resolve deeper nodes because of large rate heterogeneity between taxa. Our analyses are based on a much more comprehensive gene set, resulting in a fully resolved phylogeny that does not have long branches and is more suitable for molecular dating. Furthermore, we employed realistic models of evolution and better-curated fossil calibrations, which gives us greater confidence in our divergence time estimates. We also find that divergences of the two main lineages, Thecosomata and Gymnosomata, are placed in the Cretaceous period, dated at 129.4 (CrI, 175.8 to 103.3) and 100.7 (137.0 to 76.4) Ma, respectively. Of the fully shelled pteropods, all coiled species share a most recent common ancestor at 107 (145.5 to 85.1) Ma and the uncoiled species at 99.1 (134.7 to 78.2) Ma (Fig. 2*A*). These estimates were robust to alternative calibration schemes and model applications (Table 2 and *SI Appendix*, Figs. S3 and S4).

All major lineages of pteropods (sea butterflies and sea angels, species with coiled and uncoiled shells) were already present in the Cretaceous and thus must have survived previous major environmental changes and associated extinctions, such as the asteroid impact at the end of the Cretaceous (K–Pg, 66 Ma; refs. 47 and 48) and the Paleocene–Eocene Thermal Maximum (PETM, 56 Ma; refs. 16, 49, and 50; Fig. 2). Many pelagic groups disappeared during the well-known mass extinction at the K–Pg boundary (51, 52), and, though it is difficult to pinpoint a specific cause of these changes (17), recent evidence from boron isotopes in foraminifers shows that rapid surface ocean acidification was associated with the impact (53). The PETM also had an impact on marine calcifiers, as shown by a rapid decrease in calcium carbonate content in marine sediments (54) and the recording of one of the largest extinctions among deep-sea benthic foraminifers (49, 55). Planktonic calcifiers were also affected during the PETM, with shifts in species assemblages and changes in species abundance of calcareous nanoplankton, but no clear signal of increased extinction (56–59). Although we show that major pteropod lineages survived the K–Pg and PETM, this does not mean that pteropods were unaffected by these crises. It may be that many lineages were lost that have not been recovered in the fossil record and which cannot be sampled from extant populations. Interestingly, however, the pteropod fossil record shows a sudden increase from a single recorded species pre-PETM (Thanetian, *Heliconoides mercinensis*) to 33 species post-PETM (Ypresian; Fig. 2*C* and Dataset S1). This led Janssen et al. (2016; ref. 60) to suggest that rapid environmental changes during the PETM may have had a triggering effect on pteropod evolution.

### Evolutionary History of Pteropods.

Our estimated divergence times for the major pteropod groups precede by far their oldest known fossils (Fig. 2*A*). The largest discrepancies are found for Gymnosomata and Pseudothecosomata, for which the oldest fossils are from the Chattian (probably a *Clione* sp., 28 to 23 Ma) and Chattian–Burdigalian periods (*Peracle amberae*, 28 to 16 Ma), respectively (ref. 41 and Dataset S1). This discrepancy is not surprising, as these groups are characterized by reduced shells or are shell-less as adults, and their microscopic (larval) shells remain mostly uncharacterized. The pteropod fossil record is generally affected by strong taphonomic bias, as their delicate aragonitic shells preserve poorly. Furthermore, micropaleontologists have traditionally focused on calcitic planktonic calcifiers (coccolithophores, foraminifers) rather than aragonite-producing ones, such as pteropods. This trend is illustrated by plotting the reported diversity of pteropod species through time (Fig. 2 *B* and *C*), showing a sharp increase in the number of recent species (165 species) compared to previous periods (24 species for the Pleistocene and a median of 31.5 species for the preceding Neogene periods). Since pteropods are considered promising as new proxy carriers in paleoceanography, recording surface ocean temperature and carbonate ion concentrations (61, 62), this renewed interest will hopefully result in a reappraisal of their fossil record in the coming years.

Surprisingly, we find similar Cretaceous origins for the coiled (Limacinoidea) and uncoiled (Cavolinioidea) shelled pteropods even though the fossil record for coiled shells extends much further (Campanian, late Cretaceous) than for uncoiled shells (Ypresian, early Eocene). The species with straight, bilaterally-symmetric shells were thought to have derived from coiled ancestors (*Altaspiratella*) that show a trend of despiralization in the fossil record starting during the early Ypresian and giving rise to the oldest representatives of Cavolinioidea (*Camptoceratops* and *Euchilotheca*; first suggested by Boas 1886; ref. 63 and reviewed in ref. 24). It is interesting to note that shell microstructure differs fundamentally between coiled and uncoiled pteropods. Uncoiled species have an outer prismatic layer and a thick inner layer with a unique helical microstructure (64, 65), whereas coiled shells have simple prismatic and crossed lamellar microstructures (21), as found in the outgroup *Aplysia* (66). Examination of shell microstructures of fossil species could shed further light on the phylogenetic placement of these fossils. For instance, the fossil *Camptoceratops priscus* from the early Eocene was found to have helical microstructure resembling that in extant Cavolinioidea (67). A recent report by Garvie et al. (68) of potentially much older uncoiled pteropod-like fossils from Mesozoic and Paleocene rocks of the southern United States found that their microstructure is, in contrast, crossed-lamellar. If future analyses show that these fossils are indeed pteropods, despiralization must have occurred multiple times throughout their evolutionary history. Reports of Cretaceous pteropods remain extremely rare despite considerable paleontological effort, which suggests that pteropods were not abundant and/or very poorly preserved during this period. Notably, Cretaceous sediments are often dominated by limestones, which are unsuitable for the preservation of thin-walled aragonitic pteropod shells (24).

We do not see a clear trend toward gradual loss of shells in our phylogeny, though shelled groups have earlier origins than unshelled ones. This hypothesis should be further assessed in future work by sampling more species belonging to the elusive Pseudothecosomata, with species ranging from fully shelled (*Peracle* spp.) to partially shelled (*Cymbulia*, *Corolla*, *Gleba* spp.) and even entirely unshelled as adults (*Desmopterus* spp.). In our phylogeny, we only included one *Peracle* species possessing an external coiled calcareous shell and one *Cymbulia* species bearing a gelatinous “pseudoconch.” Since the pteropod outgroups are benthic gastropods (Aplysiida, Cephalaspidea) with mostly coiled or reduced shells, the ancestor of pteropods most likely lived on the seafloor and had a coiled shell (28, 30). Some authors have proposed that pteropods evolved in a neotenic fashion, with larvae of benthic gastropods becoming sexually mature and living their full life cycle in the open water column, because of “juvenile” shell characters such as the sinistral spiral and aragonitic shell structure (69, 70). However, this hypothesis was questioned by Jägersten (1972; ref. 71), who argued that the foot—an adult feature—played a decisive role in the transition from a benthic to a holoplanktonic existence by evolving from a creeping organ to swimming fins. It is interesting to note that a similar hypothesis was proposed for the only other extant group of holoplanktonic gastropods, the heteropods (Pterotrachoidea). For this group, the earliest fossil, *Coelodiscus minutus*, occurs in the early Jurassic and represents the oldest known holoplanktonic gastropod (72). Colonization of the open water column required numerous adaptations in both groups of holoplanktonic gastropods independently: pteropods belonging to subclass Heterobranchia and heteropods belonging to subclass Caenogastropoda.

### Fate of Pteropods from Cretaceous to Anthropocene.

Pteropods evolved in the early Cretaceous and thus were contemporaries of other major calcifying groups in the open ocean, such as ammonites and foraminifers (Fig. 2*B*). Based on their fossil records, Tajika et al. (2018; ref. 23) suggested that planktonic gastropods filled the ecological niche left empty by juvenile ammonites after their extinction at the end of the Cretaceous. However, this seems less likely given our earlier estimated origins of both sea butterflies (thecosomes) and sea angels (gymnosomes). Instead, we propose that sea angels evolved as specialized predators of sea butterflies during the Cretaceous, perhaps in an evolutionary arms race (73) in which thecosomes evolved stronger and ever more sophisticated shells (e.g., *Diacria*, *Cavolinia*), and gymnosomes evolved adaptations for prey capture and extraction, specific to the shell shapes of their prey. For instance, *Clione* feed exclusively on certain *Limacina* species by manipulating the coiled shells with their flexible buccal cones and extracting the prey with their hooks, while *Pneumodermopsis* feed on long and straight-shelled *Creseis* species using their long and flexible proboscis for prey extraction (21, 74). We thus consider it plausible that different groups of pteropods coevolved during a period that is referred to as the Marine Mesozoic Revolution. In this major evolutionary episode, a series of ecological shifts took place on the seafloor because of the evolution of powerful, relatively small, shell-destroying predators, including a massive radiation of predatory gastropods in the Early Cretaceous (75, 76). This forced benthic gastropods to develop heavily armored shells and perhaps also to escape into the open water column, as was suggested for earlier geological periods (72, 77). Furthermore, during the mid-late Cretaceous, major changes in ocean circulation, stratification, and nutrient partitioning took place that were favorable for plankton evolution, particularly for planktonic calcifiers (78).

Although the open ocean may have been a refuge for gastropods in the early Cretaceous, it is an increasingly challenging habitat to survive in during the Anthropocene. Planktonic gastropods have evolved thin, fragile shells of aragonite that are sensitive to ocean acidification, and most species live in surface waters where CO_2_ is absorbed by the ocean. Incubation experiments with shelled pteropods mimicking future ocean conditions have shown that elevated pCO_2_ and undersaturated aragonite conditions cause decreased calcification rates (79), shell dissolution (80), increased mortality (10, 81), and differential expression of genes involved in neurofunction, ion transport, and shell formation (13, 15). However, such experiments have primarily assessed phenotypic responses in short-term, single-generation studies, and thus cannot take into account the abilities of organisms to acclimate or adapt to changing conditions over longer timescales. The geological record, alternatively, can provide insight into long-term evidence of ocean acidification and the associated responses of marine calcifiers. However, the fossil record of pteropods is far from complete, and we need to rely on estimates of molecular divergence times to resolve the tempo and pattern of their evolution. The fact that pteropods have survived previous episodes of ocean acidification, such as during the PETM, does not, however, mean that they are infinitely resilient to current changes. Current rates of carbon release from anthropogenic sources are at least an order of magnitude higher than we have seen for the past 66 million years (82). Although our results suggest resilience of pteropods to past ocean acidification, it is unlikely that they have ever, during their entire evolutionary history, experienced global change of the magnitude and speed that we see today.

## Materials and Methods

### Sample Collection.

Pteropod specimens were collected on Atlantic Meridional Transect (AMT) cruises 22 and 24 (2012, 2014) using 0.71-m-diameter bongo and RMT1 nets towed obliquely between a median of 305 m depth and the sea surface (200-µm and 333-µm nets; *SI Appendix*, Table S1). Animals were sorted from bulk plankton and identified live under a light microscope to species level (when possible) based on the most recent taxonomic information, following the World Register of Marine Species (www.marinespecies.org). Specimens were preserved in RNALater (Invitrogen) and flash-frozen in liquid nitrogen, with storage at −80 °C.

### Transcriptome Sequencing and Filtering.

RNA was extracted using RNAeasy micro or mini kits (Qiagen) after homogenization with a TissueLyser (Qiagen). Because of the small size of individual specimens for some species (e.g., *Heliconoides inflatus*, *Limacina bulimoides*), the pooling of individuals (up to a maximum of 10) was needed to obtain sufficient RNA for sequencing (*SI Appendix*, Table S1). RNA quantity was determined by fluorometry using a Qubit (Invitrogen), and RNA integrity was assessed using the Xperion system (Bio-Rad). RNA-seq libraries were prepared using the TruSeq RNA Library preparation kit (Illumina), and between 12 and 33M paired-end reads per sample were sequenced for 100 cycles on a HiSeq2000 platform at the Wellcome Trust Centre for Human Genetics (Oxford). A total of 2.3 to 6.6 Gb of DNA sequence data was obtained from each of 22 samples, ensuring accurate de novo transcriptome assemblies (*SI Appendix*, Table S2). Reads were deposited to the SRA under the BioProject accession PRJNA591100. After quality assessment with Fastqc (83), reads were trimmed using sickle (84) and subsequently assembled using Trinity (v2.3.2) with default parameters and a k-mer of 25 (85).

To avoid cross-contamination between samples sequenced on the same Illumina lanes (possibly due to index “hopping”), we applied a filtering procedure based on relative transcript expression across datasets, similar to the one implemented in Croco (86). Briefly, for the assembled transcriptome of each dataset, we measured expression level using Kallisto (v0.42.4) (87) in each of the multiple datasets sequenced simultaneously. We calculated the ratio of TPMs between the putatively contaminating datasets and the original dataset and excluded transcripts with a contaminant enrichment greater than twofold enrichment and a minimal count lower than two in the original dataset. Across datasets, a median of 6% of transcripts were excluded on the first criterion and a median of 26% on the second.

De novo assembled transcriptomes usually include a high degree of redundancy, as alternative transcripts derived from the same genes are distinguished in the assembly. As this might constitute a problem for orthology assignment, we clustered transcripts based on the fraction of remapped reads that they share. To do so, we mapped reads from transcriptomes back to transcripts using Bowtie2 enabling up to 50 multimappers (-k 50) and processed the resulting alignments with Corset (v1.06) (88). Then, we estimated transcript expression using Kallisto (v0.43.1) (87), and we selected the most highly expressed transcript with each Corset cluster as a reference transcript for subsequent steps. The best open reading frame (ORF) for each of these selected transcripts was predicted using TransDecoder (v5.0.2) using a BLAST against a version of the UniProt Knowledgebase limited to metazoan taxa (e-value 10^−5^) (89).

### Phylogenetic Analyses.

We conducted orthology inference using the OMA package (v2.3.0), which performs Smith–Waterman alignment and identifies orthologs based on evolutionary distance (90). We selected single-copy orthologs (OMA groups) represented by at least one of the three outgroup taxa (*A*. *californica*, *H*. *antillarum*, or *P*. *angasi*) and at least half of the 28 ingroup taxa. These cutoffs yielded 2,654 single-copy orthologs suitable for phylogenetic analysis. For each ortholog family, protein sequences were aligned using Mafft, and poorly aligned regions trimmed using Trimal (91) for sites with a gap in more than 75% of taxa (-gt 0.15) and applying a low minimum similarity threshold (-st 0.001). The concatenation of these 2,654 orthologous genes yields a supermatrix of 834,394 positions with a total fraction of 35.75% missing data. We performed maximum-likelihood reconstruction with ExaML (v3.0.17) using a partitioned site-homogeneous model (one partition per ortholog) and an LG+Γ_4_ model (92). Node support was calculated using 100 bootstrap replicates inferred with independently generated starting trees (Fig. 1). To compare results with a site-heterogeneous model, we used Bayesian inference and a reduced dataset (because the complete dataset would be computationally intractable). We selected a subset of 200 genes which showed the maximal average bootstrap support when analyzed independently (using RAxML v8.1.18; LG+Γ_4_ model and 100 rapid bootstraps) (93, 94). The concatenation of these 200 genes generated a 108,008-amino acid alignment. This alignment was analyzed using PhyloBayes-MPI (v1.6) assuming a CAT + GTR+Γ_4_ model with chains run for more than 1,500 generations with 500 discarded as burn-in. Chain convergence was checked, and maxdiff found at 0 (95).

### Molecular Divergence Time Inference.

We used PhyloBayes (v4.1c) to infer molecular divergence times using the reduced 200-gene supermatrix and the topology from Fig. 1 (ML analysis) (96). We used the CAT + GTR+Γ_4_ model of sequence evolution because it is the most appropriate for dealing with across-site heterogeneities while minimizing long-branch attraction. We evaluated three relaxed clock models, the lognormal autocorrelated process, the CIR process, and the uncorrelated gamma multipliers (reviewed in ref. 97; *SI Appendix*, Fig. S2). Using a 10-fold cross-validation procedure, we found a best fit of the CIR relaxed clock model and of the birth–death prior on divergence times with soft bounds of 0.05 on calibration points (Fig. 2 and *SI Appendix*, Fig. S2), and therefore chose these parameters for our main divergence time inferences (Fig. 2*A*). We also applied a gamma prior on the root with a mean age of 150 Myr and SD of 70 Myr. This root prior was meant to be broad and was chosen based on the oldest known crown heterobranch (i.e., *Sinuarbullina*, belonging to the extant superfamily Acteonoidea dated 240 Ma; refs. 98 and 99) and species referred to as *Akera* (*Akera mediojurensis* and *A*. *neocomiensis* dated at 163 to 166 Ma and 133 Ma, respectively; refs. 42 and 100 and *SI Appendix*). The root prior was tested by running the analysis without calibrations to verify that the priors did not overconstrain the estimated ages. This analysis returned a posterior distribution on the root of mean 154 ± 32 Myr, supporting the priors as appropriate.

### Evaluating Alternative Schemes, Models, and Priors.

We ran analyses to assess the impact of six calibration schemes, three clock models, and three different priors on divergence time estimates using PhyloBayes. We applied eight calibration points as minimum ages based on the oldest representatives for different clades of pteropods (Table 1 and Fig. 2*A*). These calibrations are based on a thorough review of all described pteropod taxa in the fossil record as well as from recent times (Dataset S1). Additionally, we assessed five alternative calibration schemes, evaluating the impact of different *Akera* calibrations and the position of the *Heliconoides* sp. calibration on divergence time estimates. Although we chose the *A*. *neocomiensis* fossil (42) from the Early Hauterivian (133 Ma) in France to date the node Pteropoda + *Aplysia*, there have been older *Akera* fossils described from the Jurassic (*A*. *mediojurensis*, 163.1 Ma; refs. 100 and 101). However, these fossils either lack some of the features of *Akera* and may not belong here or are doubtful due to poor preservation (*SI Appendix*). It is also possible that true *Akera* representatives are even younger; for example, *Akera striatella* is known from the middle Eocene, and is poorly distinguishable from the recent species *A. bullata* (45). Further research, including analysis of fossil morphological characters, is necessary to resolve the exact phylogenetic relationships of *Akera* within Aplysiidae and to Pteropoda. *Heliconoides* sp. is the oldest pteropod fossil found thus far, with a coiled shell and clearly belonging to the Limacinoidea (schemes 3, 4, and 6). However, we also tested the impact of a different placement of this fossil as the oldest Euthecosomata (schemes 1, 2, and 5). These considerations resulted in six different calibration schemes (Table 2 and *SI Appendix*, Figs. S3 and S4).

We further tested the interplay of the three clock models (UGAM, log-normal, and CIR; *SI Appendix*, Fig. S3) and three different priors on divergence time (uniform, birth–death, and Dirichlet; *SI Appendix*, Fig. S4) using the same other parameters as previously described. These analyses show that, although the overall variance around divergence time estimates increases when including different calibration schemes, clock models, and priors, the overall conclusions remain valid. All main pteropod groups (Euthecosomata, Pseudothecosomata, Gymnosomata) originate in the Cretaceous and have survived previous perturbations in the Earth’s carbon cycle during the K–Pg and PETM (*SI Appendix*, Figs. S2–S4 and Table 2).

## Supplementary Material

Supplementary File

Supplementary File

## Data Availability

Transcriptome data have been deposited in the NCBI database under BioProject accession PRJNA591100. Alignments and files generated during analyses including Bayesian sample and bootstrap replicates are available as a Zenodo dataset: doi: 10.5281/zenodo.3479131. All data are included in the article and *SI Appendix*.
