# Supplementary material for: The origin and diversification of pteropods precede past perturbations in the Earth’s carbon cycle
Source: Proc Natl Acad Sci U S A. 2020 Sep 24;117(41):25609–17. doi: 10.1073/pnas.1920918117 (PMC7568333; doi:10.1073/pnas.1920918117)
Supplement: Supplementary File [file pnas.1920918117.sapp.pdf]

SI Appendix

The origin and diversification of pteropods precede past perturbations in the Earth's carbon cycle

Katja T.C.A. Peijnenburg, Arie W. Janssen, Deborah Wall-Palmer, Erica Goetze, Amy Maas, Jonathan A. Todd, Ferdinand Marlétaz

www.pnas.org/cgi/doi/10.1073/pnas.1920918117

Table S1. Sampling details of pteropod taxa for which new transcriptome data were collected.

Species	Taxonomic group	Latitude	Longitude	Collection date	Max depth of sample (m)	Net type, mesh size	number of individuals used in RNAextraction
Cavolinia inflexa	Euthecosomata	30° 35.9 S	25° 48.3 W	22/10/14	163	Bongo, 200µm	2
Clio cuspidata	Euthecosomata	39° 24.6 N	22° 28.6 W	01/10/14	274	Bongo, 200µm	1
Clio pyramidata	Euthecosomata	18° 19.9 N	28° 48.5 W	07/10/14	91	Bongo, 200µm	1
Creseis acicula	Euthecosomata	20° 51.3 S	25° 04.7 W	20/10/14	309	Bongo, 200µm	3
Creseis virgula	Euthecosomata	18° 19.9 N	28° 48.5 W	07/10/14	91	Bongo, 200µm	3
Cuvierina atlantica	Euthecosomata	28° 21.7 S	25° 27.3 W	11/11/12	301	Bongo, 333µm	1
Cymbulia sibogae	Pseudothecosomata	11° 22.2 N	27° 19.9 W	09/10/14	70	Bongo, 200µm	1
Diacavolinia longirostris	Euthecosomata	6° 37.1 N	28° 19.0 W	29/10/12	404	Bongo, 333µm	1
Diacria danae	Euthecosomata	24° 27.4 S	25° 02.5 W	21/10/14	323	Bongo, 200µm	2
Diacria trispinosa	Euthecosomata	24° 03.4 N	29° 54.5 W	06/10/14	258	Bongo, 200µm	1
Heliconoides inflatus (ATL)	Euthecosomata	4° 03.0 N	26° 27.8 W	30/10/12	388	Bongo, 333µm	10
Heliconoides inflatus (S ATL)	Euthecosomata	37° 53.6 S	28° 44.2 W	25/10/14	372	Bongo, 200µm	6
Hyalocylis striata	Euthecosomata	7° 17.1 N	26° 29.5 W	11/10/14	329	Bongo, 200µm	1
Limacina antarctica	Euthecosomata	44° 37.4 S	40° 41.5 W	29/10/14	350	Bongo, 200µm	5
Limacina bulimoides	Euthecosomata	24° 27.4 S	25° 02.5 W	21/10/14	323	Bongo, 200µm	5
Limacina lesueuri	Euthecosomata	14° 12.4 N	27° 55.7 W	09/10/14	305	Bongo, 200µm	4
Limacina trochiformis	Euthecosomata	25° 29.1 S	25° 00.0 W	09/11/12	153	RMT1, 333µm	1
Peracle reticulata	Pseudothecosomata	14° 12.4 N	27° 55.7 W	09/10/14	305	Bongo, 200µm	3
Pneumoderma violaceum	Gymnosomata	10° 46.8 N	27° 12.4 W	10/10/14	323	Bongo, 200µm	1
Pneumodermopsis sp.	Gymnosomata	31° 20.4 S	26° 06.0 W	23/10/14	330	Bongo, 200µm	1
Spongiobranchaea australis	Gymnosomata	41° 28.6 S	33° 51.5 W	27/10/14	228	Bongo, 200µm	1
Styliola subula	Euthecosomata	30° 35.9 S	25° 48.3 W	22/10/14	163	Bongo, 200µm	2

Table S2. Details of 28 transcriptome datasets used in phylogenomic analyses. N_reads is the number of sequenced paired-end reads, N_pept is the number of predicted peptides among transcripts. N_OG is the number of selected single-copy orthologues recovered for this species. P_miss_OG is the percentage of missing single-copy orthologues for the taxon, P_gaps is the fraction of gaps and P_miss_tot is the total fraction of missing data in the alignment for the taxon (gaps and missing genes).

Species	Order	Taxonomic group	accession	N_reads	N_pept	N_OG	P_miss_OG	P_gaps	P_miss_tot
Aplysia californica	Aplysiida	Outgroup	ftp.broadinstitute	-	92469	2616	1.09	1.6	2.69
Cavolinia inflexa	Pteropoda	Euthecosomata	SRR10527262	14107853	20521	1729	35.51	2.17	37.68
Clio cuspidata	Pteropoda	Euthecosomata	SRR10527261	15473224	21307	1222	56	1.05	57.05
Clio pyramidata	Pteropoda	Euthecosomata	SRR10527250	15596539	24705	1665	39.3	2.12	41.42
Clione antarctica	Pteropoda	Gymnosomata	SRR1505107	20282761	19813	1915	29.53	2.62	32.16
Pneumodermopsis sp.	Pteropoda	Gymnosomata	SRR10527247	18094803	18984	1396	51.35	1.24	52.59
Creseis acicula	Pteropoda	Euthecosomata	SRR10527246	12027779	27003	1806	33.19	2.82	36.01
Creseis virgula	Pteropoda	Euthecosomata	SRR10527245	14105581	26726	1824	34.12	3.06	37.18
Cuvierina atlantica	Pteropoda	Euthecosomata	SRR10527244	28558114	20994	1966	28.34	2.99	31.33
Cymbulia sibogae	Pteropoda	Pseudothecosomata	SRR10527243	13430464	14494	1166	57.8	1.83	59.63
Diacavolinia longirostris	Pteropoda	Euthecosomata	SRR10527242	14469630	21220	1582	44.76	2.22	46.98
Diacria danae	Pteropoda	Euthecosomata	SRR10527241	25218341	36533	1673	38.09	1.23	39.32
Diacria trispinosa	Pteropoda	Euthecosomata	SRR10527260	13123731	16800	682	76.48	0.87	77.36
Haminoea antillarum	Cephalaspidea	Outgroup	SRR1505111	-	39489	1821	35.68	5.68	41.36
Heliconoides inflatus (ATL)	Pteropoda	Euthecosomata	SRR10527259	33421222	20283	1109	66.97	2.08	69.05
Heliconoides inflatus (S ATL)	Pteropoda	Euthecosomata	SRR10527258	18330623	33123	1925	29.03	2.73	31.76
Heliconoides inflatus (MED)	Pteropoda	Euthecosomata	PRJNA312154	-	25016	2434	7.83	2.2	10.03
Hyalocylis striata	Pteropoda	Euthecosomata	SRR10527257	20300621	33352	2360	10.42	2.23	12.65
Limacina bulimoides	Pteropoda	Euthecosomata	SRR10527256	18046287	28764	1799	34.54	2.81	37.35
Limacina antarctica	Pteropoda	Euthecosomata	SRR10527255	19486196	28954	2221	15.92	2.07	17.99
Limacina lesueuri	Pteropoda	Euthecosomata	SRR10527254	14648179	24430	1793	33.53	2.1	35.64

Limacina retroversa	Pteropoda	Euthecosomata	PRJNA260534	-	40077	2376	10.62	2.53	13.15
Limacina trochiformis	Pteropoda	Euthecosomata	SRR10527253	14412701	35829	2004	25.99	2.75	28.74
Peracle reticulata	Pteropoda	Pseudothecosomata	SRR10527252	17876973	27998	1776	35.18	2.43	37.61
Philine angasi	Cephalaspidea	Outgroup	SRR1505129	11881480	33219	2037	23.05	5.81	28.87
Pneumoderma violaceum	Pteropoda	Gymnosomata	SRR10527251	16362957	24989	2148	17.78	2.21	19.99
Spongiobranchaea australis	Pteropoda	Gymnosomata	SRR10527249	18191206	20998	1621	40.7	1.66	42.36
Styliola subula	Pteropoda	Euthecosomata	SRR10527248	16115592	29755	2062	20.76	2.33	23.09

SI Dataset 1. Overview of all pteropod taxa (fossil and recent) through time. Information is provided on taxonomy, numerical age, dating method and biogeography. FOD is First Occurrence Datum and LOD is Last Occurrence Datum.

Figure S1. Phylogenomics resolves evolutionary relationships of pteropods. Euthecosomes (fully shelled species, green) and pseudothecosomes (ranging from fully shelled to unshelled species, orange) are recovered as sister clades for the first time in a molecular analysis restoring the Thecosomata ('sea butterflies') as a natural group. Thecosomata and Gymnosomata ('sea angels', purple) are monophyletic sister clades congruent with traditional morphology-based views. The superfamilies Cavolinioidea with uncoiled shells and Limacinoidea with coiled shells are also recovered as monophyletic sister clades. Bayesian phylogeny of 25 pteropod taxa, plus 3 outgroups assuming a CAT+GTR+Γ_4 model using Phylobayes-MPI. The dataset was comprised of 200 selected genes (see Methods), concatenated as 108,008 amino acid positions. All nodes received maximal posterior probabilities.

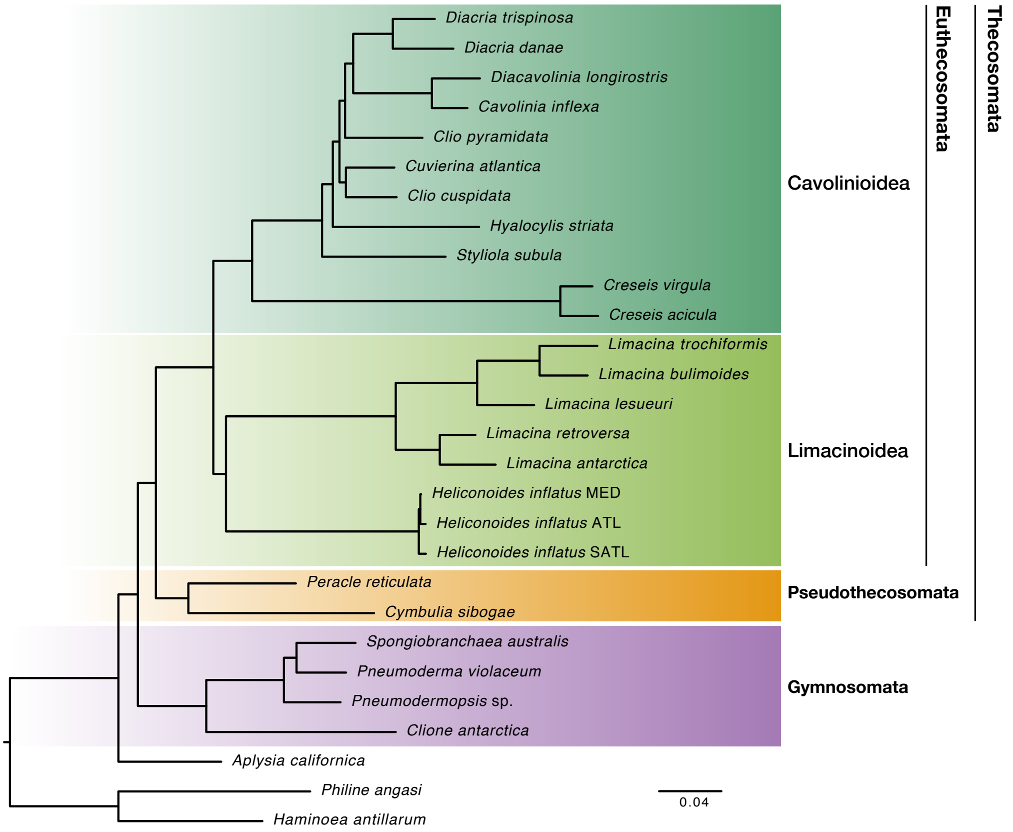

Figure S2. **Divergence times for major pteropod clades under different clock models were not markedly different.** Bayesian phylogenies to infer molecular divergence times using the reduced 200 genes supermatrix, the topology of Figure 1 and a CAT+GTR+Γ_4 model of sequence evolution (see Materials and Methods) with three different clock models: the CIR process (CIR, see also Figure 2*A*), the lognormal autocorrelated process (Log-normal), and the uncorrelated gamma multiplier process (UGAM).

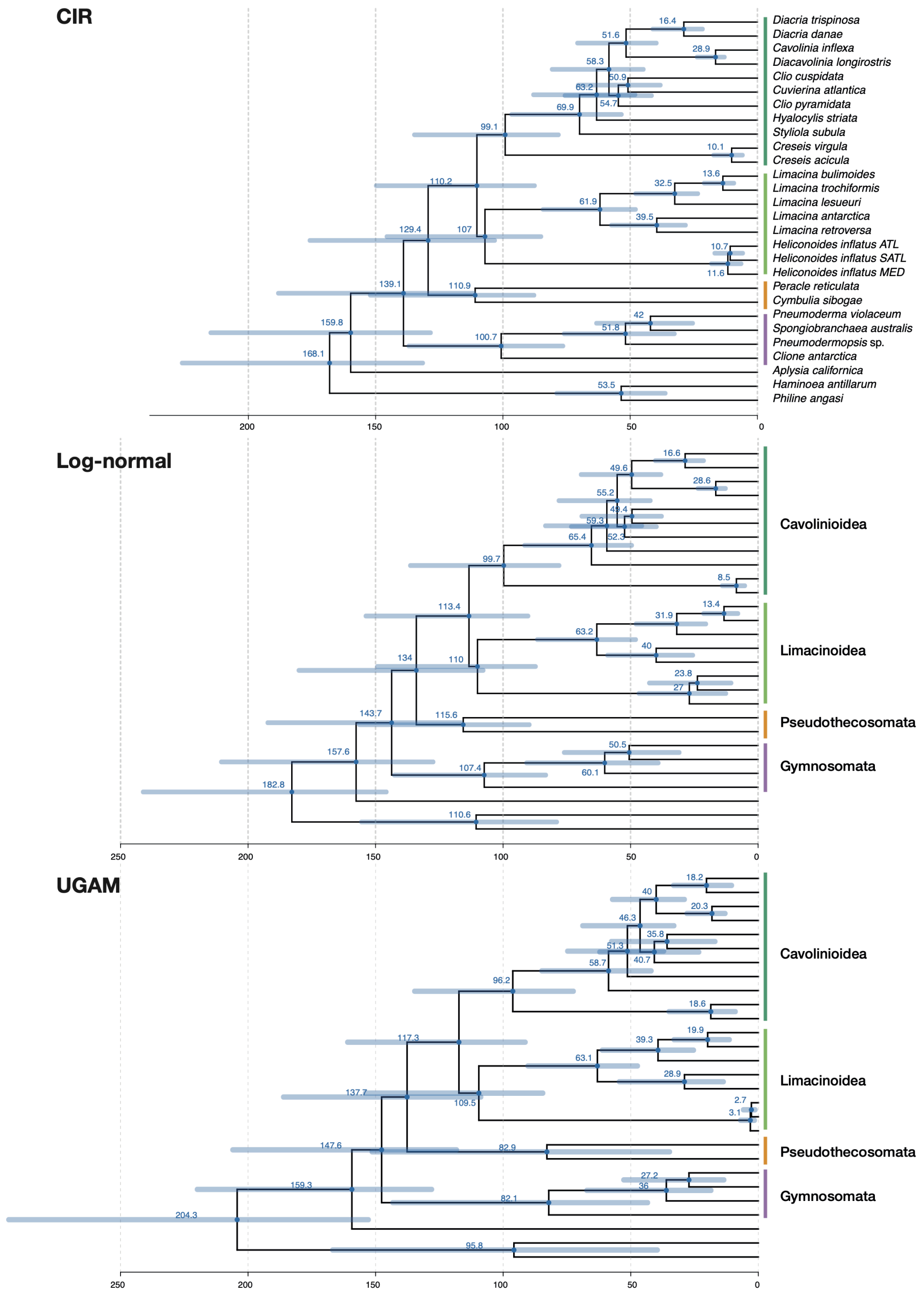

Figure S3. Divergence time estimates for major pteropod clades under six calibration schemes and three different clock models: the CIR process (CIR), the lognormal autocorrelated process (Log-normal) and the uncorrelated gamma multiplier process (UGAM). These different models were all applied on the reduced 200 gene supermatrix (108,000 amino acids) with the topology from Figure 1 (ML analysis), a CAT+GTR+Γ_4 model of sequence evolution, a gamma prior for the root age as 150 $\pm$ 70 Myr and soft bounds on calibration points (see Material and Methods for more details). The six calibration schemes assessed the impact of different *Akera* calibrations and the position of the *Heliconoides* sp. calibration on divergence time estimates, as follows: s1: *Akera mediojurensis*, 163.1 Ma; *Heliconoides* sp. as oldest Euthecosomata; s2: *Akera neocomiensis*, 133 Ma; *Heliconoides* sp. as oldest Euthecosomata; s3: *Akera mediojurensis*, 163.1 Ma; *Heliconoides* sp. as oldest Limacinoidea; s4: *Akera neocomiensis*, 133 Ma; *Heliconoides* sp. as oldest Limacinoidea; s5: no *Akera* calibration; *Heliconoides* sp. as oldest Euthecosomata; s6: no *Akera* calibration; *Heliconoides* sp. as oldest Limacinoidea.

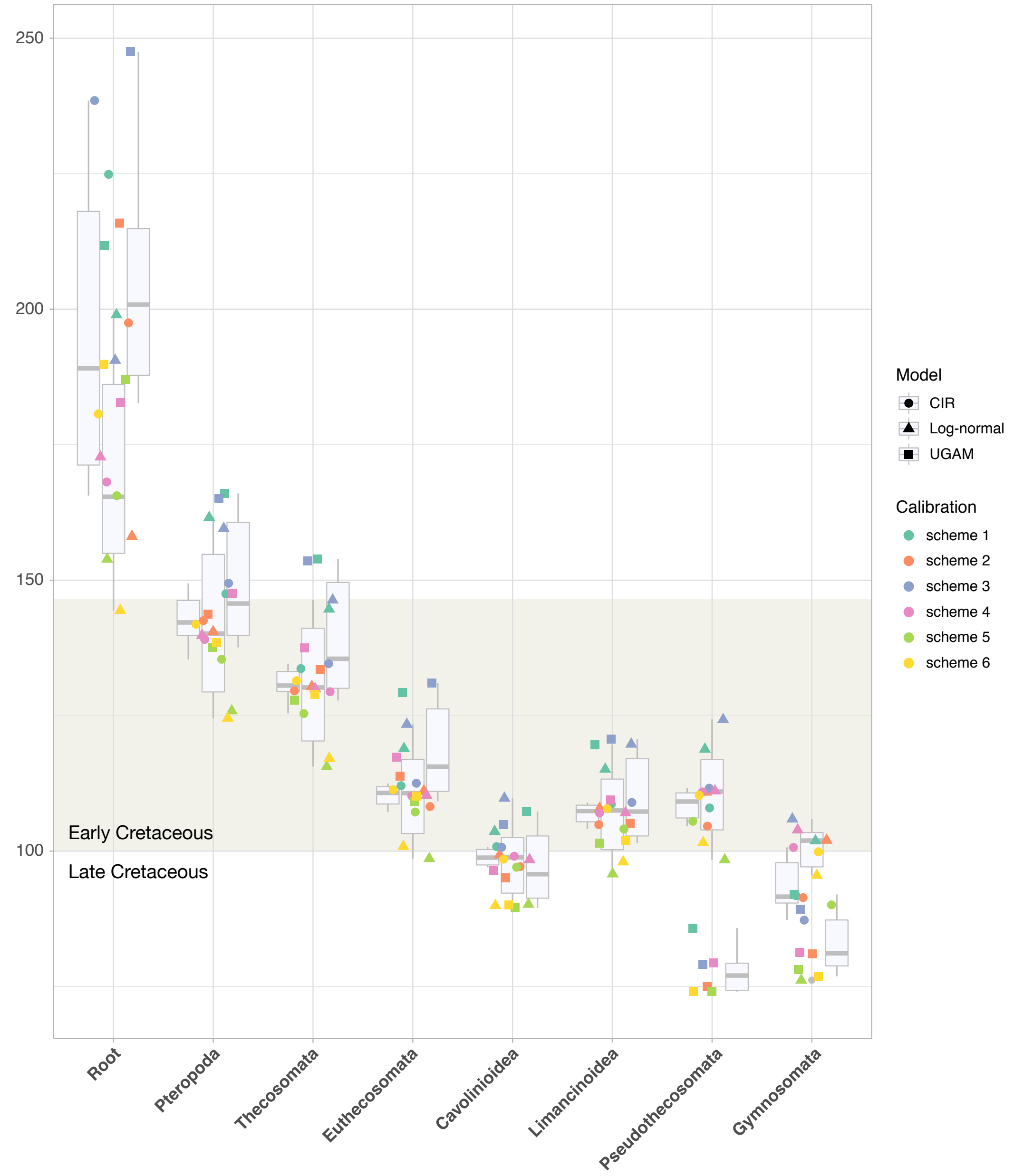

Figure S4. Divergence time estimates for major pteropod clades under six calibration schemes and three different priors on divergence time: uniform, birth-death, and Dirichlet (see Phylobayes manual). These different models were all applied on the reduced 200 gene supermatrix (108,000 amino acids) with the topology from Figure 1 (ML analysis), a CAT+GTR+Γ_4 model of sequence evolution, a gamma prior for the root age as 150 $\pm$ 70 Myr and soft bounds on calibration points (see Material and Methods for more details). The six calibration schemes assessed the impact of different *Akera* calibrations and the position of the *Heliconoides* sp. calibration on divergence time estimates, as follows: s1: *Akera mediojurensis*, 163.1 Ma; *Heliconoides* sp. as oldest Euthecosomata; s2: *Akera neocomiensis*, 133 Ma; *Heliconoides* sp. as oldest Euthecosomata; s3: *Akera mediojurensis*, 163.1 Ma; *Heliconoides* sp. as oldest Limacinoidea; s4: *Akera neocomiensis*, 133 Ma; *Heliconoides* sp. as oldest Limacinoidea; s5: no *Akera* calibration; *Heliconoides* sp. as oldest Euthecosomata; s6: no *Akera* calibration; *Heliconoides* sp. as oldest Limacinoidea.

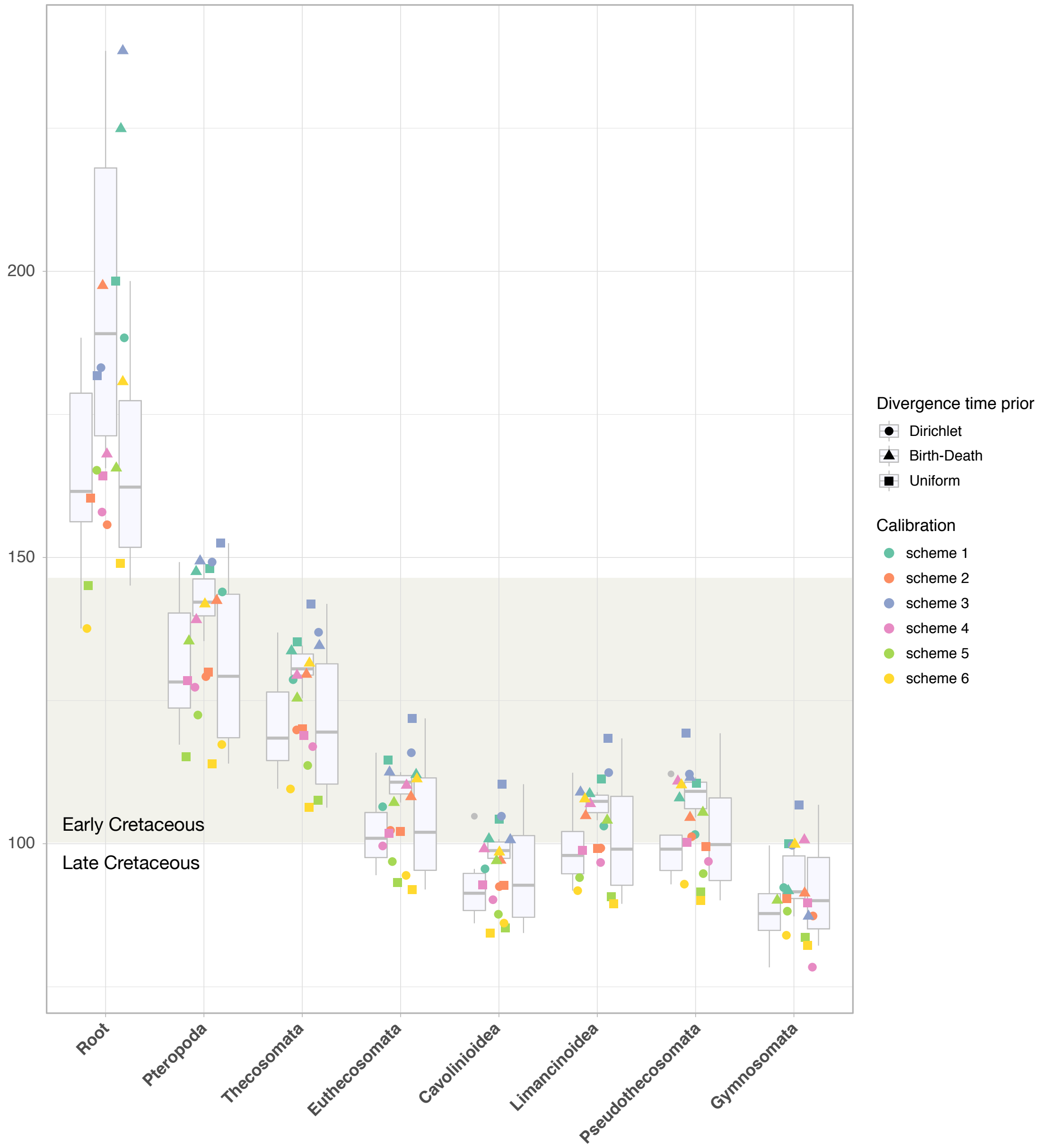

Assessing different schemes, models and priors

Overall, as expected, divergence time estimates of major groups of pteropods are older when the older *Akera* calibrations are used (s1 and s3) and younger when this node is left unrestricted (s5 and s6). Similarly, age estimates are older when *Heliconoides* sp. is used to calibrate the Euthecosomata node (s1, s2 and s5) compared to the Limacinoidea node (s3, s4, s6) (see also Table 2). Nevertheless, the divergence time estimate of Pteropoda is stable across different calibration schemes, ranging from 135 Ma (s5) to 149 Ma (s3), and is thus firmly placed in the Early Cretaceaous. In Figure 2 we depict the results from calibration scheme 4 with the divergence of Pteropoda estimated at 139.1 Ma with 95% credibility interval 111.4-188.2 Ma.

Examining the impact of different clock models (Fig. S3) shows that the variance around average age estimates based on different calibration schemes and the CIR model are generally smaller than for the other clock models (-Ln and UGAM). The age estimates for the older nodes (e.g. Pteropoda, Thecosomata, Euthecosomata) are more similar across the different clock models compared to the more recent clades (Pseudothecosomata, Gymnosomata). The biggest difference is for the age of Pseudothecosomata, which is estimated for calibration scheme 4 and a birth-death prior as 111 and 79 Ma, for the CIR and UGAM model, respectively.

Divergence time estimates using the birth-death prior are generally older than estimates based on the uniform or Dirichlet priors (Fig. S4). Nevertheless, average age estimates based on six calibration schemes for the major groups Thecosomata and Gymnosomata did not differ widely comparing a birth-death prior (131.2$\pm$3.3 and 93.3$\pm$5.2, respectively) with a uniform prior (121.7$\pm$14.4 and 92.1$\pm$ 9.6, respectively).

A critical appraisal of the fossil record of *Akera* and the choice of calibration dates
[by J. A. Todd]

The Aplysiida (=Anaspidea) has been identified as the sister clade to the Pteropoda based on both morphological and molecular analyses (1, 2) and this node is used to date the origin of the Pteropoda.

Among the relatively low diversity Aplysiida, the fossil record is confined to shelled species. The Aplysioidea: Aplysiidae have fossilized internal shells known as early as the late Early Eocene (Ypresian) (*Floribella*) with a spotty record extending through to the Recent (3). However, the less reduced shells of the sister taxon to the Aplysioidea, the genus *Akera* (Akerioidea: Akeridae) has a much longer record being reported back as far as the Middle Jurassic (Bathonian) (4). However, it is not widely recognized that the taxonomy and systematics of fossil bullomorph gastropods (bubble shells) still requires extensive revision, especially Mesozoic fossils which are often incompletely preserved as recrystallized shells or partial or wholly internal moulds in carbonate lithologies. The fossil record of *Akera* itself has not been critically revised since Cossmann's work (refs. 4 and 5) in the late nineteenth century despite very active ongoing revision of basal heterobranch taxa and their systematics.

Tracey et al. (ref. 6) cited two species, *A. mediojurensis* and *A. tanganyicensis* of Middle Jurassic (Callovian) age as the oldest representatives of *Akera*, and discarded, without comment, Cossmann's uncertain taxonomic placement of the older *A.? primaeva* (Deslongchamps) from the Bathonian, Middle Jurassic. Tracey et al. (ref. 6) could only attempt very limited critical reappraisal of records in their data compilation yet this has been widely used for fossil calibration of molecular phylogenies despite it being made for a very different purpose.

To justify the calibration dates chosen here, we present notes on our taxonomic re-evaluation of fossil taxa that have been ascribed to *Akera* in the literature. Among the bubble shells, extant *Akera* has a relatively complex shell morphology compared to many basal heterobranchs and cephalaspideans and this gives us confidence in our assessments of morphology when this is well preserved in fossils. Unfortunately, most geologically old (Mesozoic) putative *Akera* are known solely from internal casts in carbonate rocks. These do not preserve characters necessary for the unambiguous identification of *Akera*, such as the morphology of the narrow inrolled inner lip and the details of the upper surface of the whorl, sutural channel and sinus, and the spire. Despite this the features that are preserved, for example the shape of the outer lip on the whorl side, allow us to assess whether associated features (e.g., evenly backwardly curved base of aperture, presence of a deep, abaperturally-positioned, sutural sinus) were present.

Direct examination of the following Recent species: *Akera bullata* (Müller, 1776), *A. soluta* (Gmelin, 1791) and *A. tenuis* (Adams, 1850) and the Eocene species *Akera striatella* (Lamarck, 1804) as well as literature on *Akera julieae* Valdés & Barwick (ref. 7) and *A. constricta* Kuroda (see ref. 8: Textfig. 3) reveal that the following shell features are shared between them all:

1) Shells are thin to very thin walled.
2) In lateral view the outer lip descends from its anterior sharp angulation arcing forwards and forming a rounded projection at or above the shell periphery at about one-fifth of whorl height.
3) Outer lip then retracts backwards much further than it extends forwards at top of shell so that base of shell comes to a point when seen in lateral view.
4) Outer lip extends around forming a wide anterior to the aperture.
5) On the inner side of the aperture the outer lip is rolled over on itself to form a thin and sharp-edged, projecting ridge that extends apically backwards into the aperture (so far one cannot see in a whole shell).
6) At its anterior extremity the outer lip forms a very sharp keel and extends back along the sharp keel a long distance before sharply sloping down and forming a rounded or more angular sinus in a deep flat-bottomed trough (canaliculation) about one-quarter of a whorl back from the outer lip.
7) The innermost part of the outer lip is flattened and bears numerous wrinkled growth increments. It narrows as it extends forward within the canaliculated region to about one-third of the distance to the outer lip. On its innermost edge it abuts the spire.
8) The inner lip then continues abaperturally as a thin parietal wash that extends diagonally down across the previous whorl to meet the raised rolled inner lip at the base of the shell.
9) The apex varies from a slightly raised spire to flat to slightly concave and has a smooth, globose heterostrophic protoconch – of which only a single whorl can be seen.
10) The shell surface is covered with very fine incised spiral ornament.
11) Sometimes wide axial ‘grooves’ seen on last whorl. These appear to be flexures of the whole shell.

This suite of characters gives a very clear idea of what to look for in the geologically oldest fossils assigned to *Akera*.

Putative Mesozoic *Akera*

Cossmann's (refs. 4 and 5) descriptions and photographic images of fossil *Akera* remain the most complete treatment and these are used as the basis for morphological re-assessment of Mesozoic taxa. No other well-preserved Jurassic or early Cretaceous species are known to have been described since, excepting *Akera tanganyicensis* Cox, 1965. The literature was supplemented by examination of available holotype material of *A. primaeva* and *A. tanganyicensis.*

Cossmann (ref. 5) noted that the Jurassic species were slightly different from living species but considered Cretaceous species to have an intermediate morphology. Jurassic species had two key features that for Cossmann permitted their assignment to *Akera*: a sutural canal with an associated strong inflection of the outer lip, and the basal (anterior) sinuosity of the aperture. As such, he felt there was a strong probability that they belonged to *Akera*. Today, these character descriptions are not detailed enough to allow attribution by themselves and a range of non-aplysiids such as the sacoglossan *Oxynoe* (see ref. 9) are known to show similar features.

Akera? primaeva (Deslongchamps, 1842)

Holotype NHMUK OR 81616.

Middle Bathonian, Middle Jurassic, France.

Preservation: a cast in carbonate. No shell preserved.

This shell has a sunken apex (more so than *Akera*) and shows strong undulating growth lines on its internal mould (just like those in *Palaeohydatina* Cossmann, 1895 but lacking the breaks seen in *P. undulata* Bean, the type species). These grooves show that it lacks a strongly retracted base. It lacks any canaliculation at the top of the whorl and therefore is unlikely to have had a sutural slit. Some calcite shell material is preserved towards the base of the inner lip – this shows a broad sweep of material extending basally (posteriorly it is broken away and not preserved) over the paries and a erect vertical 'pillar' (possibly behind which was a very narrow umbilical chink). This is very different to the inner lip of *Akera*. It seems that the more poorly preserved specimen figured by Cossmann (1896) is unlikely to give much more information.

Conclusion: There is little reason to believe that this is an *Akera*.

Akera mediojurensis (Cossmann, 1896) (p. 128; pl. 6, figs 8, 9)

Callovian-Oxfordian, Middle-Upper Jurassic, France

Preservation: Internal cast in carbonate.

Cossmann, 1896 (ref. 5): Pl. 6, fig. 9 seems to show that strong growth lines are present on the cast (like *Palaeohydatina*). It is described as having a deep notch at the periphery of the "excavation" of its spire but this is difficult to evaluate without examination. The aperture appears not to be strongly retracted at its base. Growth lines are slightly arched and feebly backwardly inclined to the anterior and feebly retracted towards the top of the whorl.

Conclusion: Without direct examination it is hard to ascertain the affinities of this taxon but the shape of the outer lip is unlike that in *Akera*. Cossmann (ref. 5) himself noted that its

taxonomic position was not certain due to the feebly curved outer lip and the deep ‘gash’ it forms at the suture.

Akera tanganyicensis Cox, 1965 (ref. 10)

Holotype NHMUK GG 10332

Callovian, Middle Jurassic, Tanzania

Preservation: This is a relatively coarsely recrystallized shell (where present) overlying a compressed marly internal mould.

This shell has a rounded top to the whorl and a slightly impressed suture though not clearly grooved. Therefore, both the canaliculation and pronounced notch are likely absent. There is no clear sign of the shape of the outer lip due to recrystallization and lack of axial flexures. Though the outer lip is broken throughout its length there is no indication of its strong retraction towards the base. The base of the inner lip shows a raised vertical pillar (like in *A. primaeva*) and some indication that there might have been a narrow umbilical chink behind it) – some shell is poorly preserved here.

Conclusion: There is little reason to believe this is an *Akera*. It may well be related to *A. primaeva* though Cox compared it to *A. mediojurensis* and to *A. truncata.*

Akera blaisiaca de Loriol, 1872 (?= *A. moreana* Buvignier, 1852)

late Kimmeridgian or Tithonian, Upper Jurassic, France

Preservation: Internal cast.

An ovoid-conical shell with its periphery lying towards the base. Its spire is almost flat and appears partly enveloped by later whorls. Growth lines are strongly backwardly directed at the top of the whorl, are slightly forwardly projecting on the side of the whorl and become backwardly curved towards the base. Aperture narrow but inner lip strongly concave according to ref. 5.

Conclusion: Cossmann (1896) considered this an undoubted *Akera*. However, his description and images do not permit its confident assignment. Its overall shape with a periphery lying the base of the shell, its less prominent sutural canaliculation, narrow aperture and lack of information on its inner lip make its affinities uncertain pending re-examination.

Akera truncata (Lennier, 1867)

late Kimmeridgian or Tithonian, Upper Jurassic, France

Preservation: Internal cast

Cossmann (ref. 5) noted that this species resembled *A. mediojuren*sis but was narrower and more cylindrical (maximum width at half-height) and lacked an excavated spire. It has a rounded top to its whorls, growth lines that arc forwards on the whorl side and become retracted towards the base where they round into the outer lip. The sutures are narrow but deeply channelled in the internal moulds.

Conclusion: It is difficult to assess this species without direct examination but its overall outline differs from *Akera* and its poor preservation as internal moulds is likely to limit interpretation.

It is possible that some of the older species discussed above might belong to a stem-*Akera* lineage but we have chosen not to speculate on what shell characters might be expected.

The first probable *Akera*

Akera neocomiensis (Cossmann, 1895)

Neocomian, Lower Cretaceous (herein revised to early Hauterivian, Radiatus zone) of Marolles, Aubes, France, see ref. 11. For absolute age (133 Ma) see ref. 12.

Preservation: Internal mould with patches of shell

Description and conclusion: As far as can be assessed from its description and small photographs, *A. neocomiensis* has the shell outline of extant *Akera*, a similar outer lip morphology and as far as can be ascertained a prominent and deep sutural channel. Notably, Cossmann (1895) considered that its morphology more closely resembled those of Tertiary species, including *A. striatella* Lamarck, which Valdés & Lozouet (2000) treated under the name *A.* cf. *bullata* as its morphology is so similar to the Recent species. Consequently, pending direct re-examination and revision we consider *A. neocomiensis* to be the earliest probable *Akera*.

SI Appendix references

1. A. Klussmann-Kolb, A. Dinapoli, Systematic position of the pelagic Thecosomata and Gymnosomata within Opisthobranchia (Mollusca, Gastropoda) – revival of the Pteropoda. *J. Zool. Syst. Evol. Res.* **44**, 118–129 (2006).

2. F. Zapata *et al.*, Phylogenomic analyses of deep gastropod relationships reject Orthogastropoda. *Proc. Roy. Soc. B* **281**, 20141739 (2014).

3. Á. Valdés, P. Lozouet, Opisthobranch molluscs from the Tertiary of the Aquitaine Basin (South-Western France), with descriptions of seven new species and a new genus. *Palaeontology* **43**, 457-479 (2000).

4. M. Cossmann, Essais de paléoconchologie comparée. Première livraison. Comptoir Geologique, Paris (1895).

5. M. Cossmann, Contribution à la Paléontologie française des terrains jurassiques. Études sur les Gasteropodes des terrains jurassiques. Mémoires de la Société Géologique de France. *Mémoires de la Société Géologique de France. Paléontologie* **14**, 1-165 (1896).

6. S. Tracey, J. A. Todd, D. Erwin, "Gastropoda" in The Fossil Record 2, M. J. Benton, Ed. (Chapman & Hall, London, 1993), pp. 131-167.

7. Á.Valdés, K. Barwick, First record of *Akera* Müller, 1776, from the eastern Pacific, with the description of a new species. *The Nautilus*, **119**, 43-49 (2005).

8. T. Habe, Hydatinidae, Bullidae, Akeridae. *Illustrated Catalogue of Japanese Shells, 3* Kyoto, Japan (1950).

9. P. J. Krug, J. S. Berriman, Á. Valdés, Phylogenetic systematics of the shelled sea slug genus *Oxynoe* Rafinesque, 1814 (Heterobranchia: Sacoglossa), with integrative descriptions of seven new species. *Invertebrate Systematics* **32**, 950-1003 (2018).

10. L. R. Cox, Jurassic Bivalvia and Gastropoda from Tanganyika and Kenya. *Bulletin of the British Museum (Natural History), Geology. Supplement* **1** (1965).

11. H. Löser, Morphology, taxonomy and distribution of the Early Cretaceous coral genus *Holocoenia* (Scleractinia) and its first record in the Caribbean. *Revista Mexicana de Ciencias Geológicas* **26**, 93-103 (2009).

12. M. Martinez, J.-F. Deconinc, P. Pellenard, L. Riquier, M. Company, S. Reboulet, M. Moiroud, Astrochronology of the Valanginian-Hauterivian stages (Early Cretaceous): Chronological relationships between the Paraná-Etendeka large igneous province and the Weissert and the Faraoni events. *Global and Planetary Change* **131**: 158-173 (2015).
